# Evaluation of left atrial strain changes in patients with breast cancer after anthracycline therapy

**DOI:** 10.1186/s43044-024-00591-2

**Published:** 2025-05-19

**Authors:** Mostafa El Mokadem, Saeed M. Shaaban, Osama Ahmed Amin

**Affiliations:** 1 Department of Cardiology, Faculty of Medicine, Beni-Suef University, Mohamed Hasen Street, Beni-Suef, 62511 Egypt; 2 Department of Clinical Oncology, Faculty of Medicine, Beni-Suef University, Mohamed Hasen Street, Beni-Suef, 62511 Egypt

**Keywords:** Breast cancer, Left atrial strain, Chemotherapy, Anthracycline, Cardiotoxicity

## Abstract

**Background:**

Cancer breast is the most common malignancy worldwide in females and is commonly treated using regimens based on anthracycline therapy. Cardiac complications related to breast cancer treatment using chemotherapy can manifest as either acute, subacute, or chronic dysfunction of the heart. Most of the signs of cardiotoxicity are subclinical. Our study aimed to evaluate left atrial strain (LAS) changes before and after anthracycline-based therapy in patients with breast cancer. One hundred and twenty patients with invasive breast cancer stages I–III eligible to receive anthracycline-based chemotherapy were enrolled in the study. We used doxorubicin 60 mg/m^2^ plus cyclophosphamide 600 mg/m^2^ every three weeks for 3–4 cycles. Every patient completed at least two echocardiograms: baseline and after each chemotherapy cycle.

**Results:**

We initially enrolled 120 patients in this observational cohort prospective study. Twenty-six patients were excluded. All LAS measures were significantly reduced at follow-up with a significant positive correlation with left ventricular global longitudinal strain. 56.4% of our patients were hypertensive, and 81% of hypertensive patients received angiotensin-converting enzyme inhibitors (ACEI). The subgroup of patients who received ACEI for treatment of HTN had a lower rate of left atrial strain changes following anthracycline therapy compared with non-ACEI-treated patients, either hypertensive or not.

**Conclusion:**

We concluded that LAS deteriorated usually after anthracycline treatment, and it predicted early chemotherapy-induced cardiotoxicity.

## Background

Breast cancer is the second most common cancer-related death in women (31%) after lung cancer and the most common malignancy among women overall [1]. Neoadjuvant or adjuvant chemotherapy is essential in early stage breast cancer as it improves survival and recurrence rates [2, 3]. Anthracycline-based regimens are widely used in neoadjuvant/adjuvant settings as studies showed that they reduce the yearly breast cancer mortality by approximately 38% compared to non-anthracycline-based cyclophosphamide, methotrexate, 5-fluorouracil regimen [4]. The term "chemotherapy-induced cardiotoxicity" describes both the direct effects of chemotherapy on the heart as well as the secondary effects that result from a thrombogenic state or changes in hemodynamic flow [5]. Anthracyclines, like doxorubicin and idarubicin, are considered essential chemotherapeutic agents. However, their efficacy in treating malignancy is limited by dose-dependent cardiotoxicity [6]. Chemotherapy for breast cancer may cause acute, subacute, or chronic cardiac side effects [5]. The majority of cardiotoxicity symptoms are not severe. Undiagnosed systolic or diastolic heart problems might result in mortality or permanent heart failure [7]. Current guidelines suggest standard measurement and then routine evaluation of cardiac function with traditional metrics like ejection fraction (EF) to identify cardiotoxicity [8]. Although echocardiography is a routine tool for ventricular assessment in patients with malignancy, it has some critical limitations. First of all, these are operator-dependent and image-quality parameters. Second, only the global left ventricular (LV) function is quantified using left ventricular EF fraction (LVEF) evaluation; the regional function is not assessed. Lastly, it should be noted that global parameters are load-dependent and unresponsive to minute variations in myocardial function. Myocardial speckles used by speckle tracking imaging (STI) can be followed frame-to-frame during cardiac cycles. Before EF dropped, individuals treated with chemotherapy had a significant decline in regional myocardial strain [9]. Both LV diastolic function and filling pressure are regulated by the left atrium (LA). In early diastole, the LA serves as a conduit for blood flowing to the LV, a pool for blood returning from the pulmonary veins, and a pump that contracts in late diastole [10, 11]. An increase in filling pressure could result from damage to one of these systems [12–14]. Recent studies show that LAS predicts LA filling pressures more accurately than the standard diastolic echocardiography parameters [13, 14]. Decreased LAS was proven beneficial in predicting cardiovascular outcomes in healthy and heart failure individuals [15].

It was hypothesized that decreased LAS would be a valuable and early predictor of subclinical LV dysfunction in patients treated with anthracycline. The early prediction of cardiotoxicity leads to the initiation of different measures for early treatment of cardiotoxicity.

Our study evaluated the LAS before and after anthracycline therapy in patients with breast cancer and assessed the different variables affecting LAS parameters.

## Methods

We initially enrolled 120 patients with breast cancer stages I–III and were eligible to receive anthracycline-based chemotherapy in the oncology department. All the patients signed informed written consent after the Research Ethical Committee approval on the 2nd of October 2022 (Approval number is FMBSUREC/02102022). All the patients completed two echocardiographic evaluations before and by the end of the chemotherapy cycles. We excluded from the study all patients below 18 years, male patients, and any underlying structural heart disease that may cause impaired LV filling, e.g., (baseline LVEF < 50% history of myocardial infarction, myocarditis, significant valvular heart diseases), previous treatment with anthracycline, concurrent treatment with dexrazoxane (cardioxane), or suboptimal echocardiographic views not appropriate for strain analysis. We used doxorubicin 60mg/m^2^ plus cyclophosphamide 600mg/m^2^ every three weeks for 3–4 cycles [16]. We excluded patients with EF less than 50%. The excluded patients followed the guidelines-directed recommendations: If EF 40–49%, we repeated echocardiography before each cycle and gave angiotensin-converting enzyme inhibitor (ACEI) or shifted to non-cardiotoxic agents (as taxanes). But, if EF < 40, we omitted anthracycline and gave a non-cardiotoxic protocol [17].

**Fig. 1:**
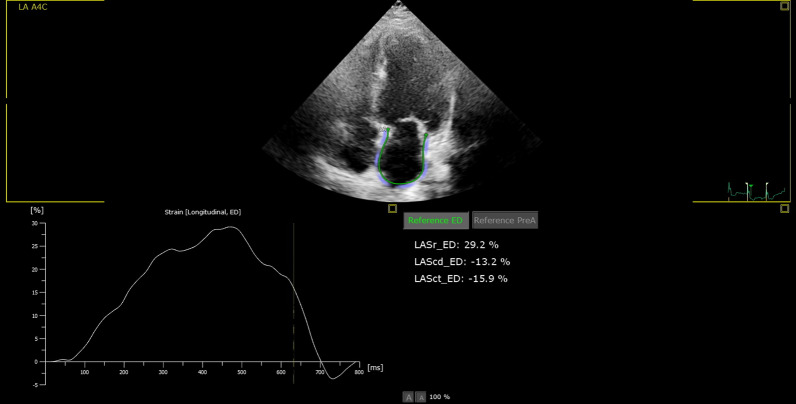
Measurement of left atrial strain measures

We completed two echocardiographic evaluations: baseline and after four chemotherapy cycles using a Philips EPIQ 7 Machine with a 3.5-MHz transducer for evaluation of left ventricular (LV) dimensions in addition to EF measured by Simpson's method [18]. During 2D echocardiography, the anteroposterior dimension of the LA was evaluated in the parasternal long-axis view at the end of LV systole. Women's average LA dimension was (2.7–3.8cm) [18]. LAS analysis was performed in the ideal apical four-chamber view image; the endocardial border was traced after the identification of regions of interest [19]. LA longitudinal strain curve was then produced (Fig. 1) to calculate conduit, reservoir, and pump functions guided by definitions previously published in past studies [20].

The normal ranges for the three components of the LA function are shown as follows [21].

- Reservoir strain 39% (95% confidence interval: 38% to 41%)
- Conduit strain 23% (95% confidence interval: 21% to 25%)
- Contractile strain 17% (95% confidence interval: 16% to 19%)

### 2D-speckle tracking echocardiography for global longitudinal strain (GLS) measurement

Two different operators performed offline analysis; both of them were blinded to baseline and follow-up cases. The regions of interest were defined by identifying the endocardial borders at the mitral annulus level and the apex of each view, and then they were divided into six segments. We considered the normal GLS range between − 17.2 and − 27.7% [22].

### Statistical methods

G_Power version Software (3.1.9.2) was used to calculate sample size based on medium effect size (0.3), overall type I error rate, (α) ≤ 0.05, and a total of 90 patients was excellent to achieve a statistical power of 80%. Scale variables were presented as mean ± SD. Qualitative values were displayed as a number (%). A paired t test was used for scale variables. We analyzed categorical data using the Chi-square test. Predictors of LAS were found using linear regression analysis. A *p* value < 0.05 was assumed to have statistical significance.

## Results

**Fig. 2:**
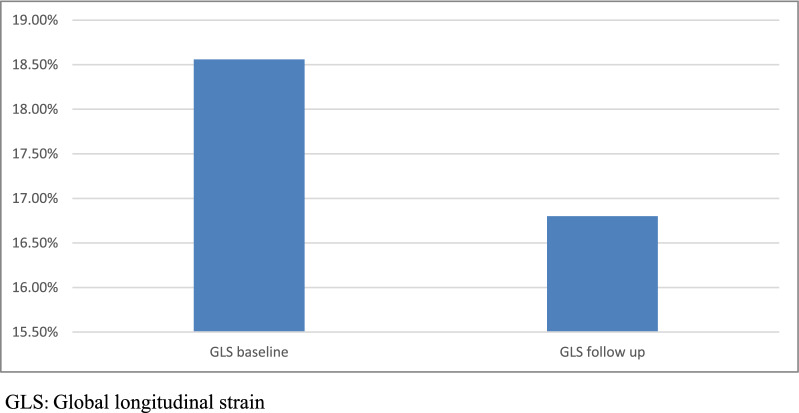
Global longitudinal strain changes before and after chemotherapy

**Fig. 3:**
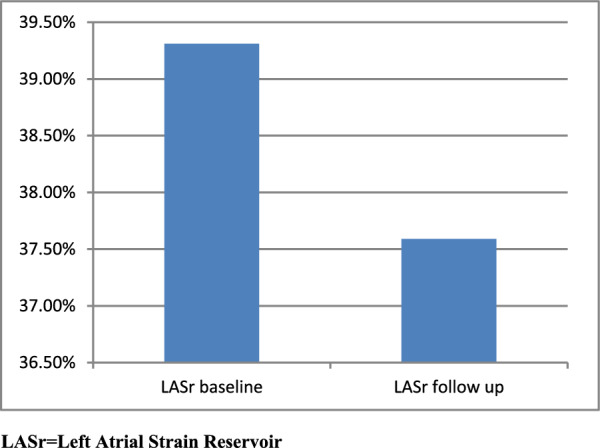
Left atrial strain changes before and after chemotherapy

We initially enrolled 120 patients in this observational cohort prospective study. However, during the treatment protocol, we excluded 26 patients after applying the exclusion criteria. Six patients were excluded because of noncompliance with the echocardiographic assessment, and 20 patients had a drop of EF below 50% before completing all the cycles. The final sample size was 94 patients. Age ranged from 21 to 71 years. None of them were smokers. Hypertensive patients were 56.4% of our enrolled patients. 81.1% of the hypertensive patients were pretreated with angiotensin-converting enzyme inhibitors (ACEIs) (70% Ramipril, 20% Perindopril, and 10% Enalapril), as shown in Table 1. ACEI-pretreated patients and non-ACEI-pretreated patients were matched regarding baseline characteristics (*P* = 0.523). All LAS and GLS measures were significantly reduced after anthracycline therapy (Figs. 2, 3). Left ventricular EF decreased significantly with a significant increase in LA dimensions after chemotherapy, as shown in Table 2. A significant positive correlation existed between LAS measures and LV GLS (Table 3). Those who developed LAS changes following the anthracycline therapy had a higher incidence of left ventricular function deterioration, indicated by a decline in LV GLS. The only independent predictor of LAS deterioration revealed by multivariate linear regression analysis is the patient subgroup (whether pretreated with ACEI or not), as shown in Table 4. Subgroup analysis revealed a significant reduction of LAS measures in non-ACEI-pretreated patients compared with those pretreated with ACEI after chemotherapy, as shown in Table 5. The LAS did not show significant changes in patients receiving different ACEIs, as shown in Table 6.

**Table 1:** Baseline characteristics of all patients under study (*n* = 94)

Characteristics	
Sex (M/F)	0/94
Age (years)	46.21 ± 12.53 (21–71)
Current smoking n (%)	0 (0%)
Hypertensives	53 (56.4%)
Antihypertensive medications	
ACEI n (% within hypertensives)	43 (81.1%)
CCB n (% within hypertensives)	6 (11.3%)
BB n (% within hypertensives)	4 (4.3%)
Diabetics	9 (9.6%)

*M* Male; *F* Female; *ACEI* Angiotensin-converting enzyme inhibitors; *CCB* Calcium channel blockers; *BB* Beta-blockers

**Table 2:** Comparison between echocardiographic results before and after anthracycline therapy

	Before chemotherapy	After chemotherapy	*P*
	(*n* = 94)	(*n* = 94)	
LVEDD cm (mean ± SD)	4.74 ± 0.50	4.70 ± 0.71	0.796
LVESD cm (mean ± SD)	3.21 ± 0.55	3.18 ± 0.40	0.680
EF% (mean ± SD)	63.05 ± 5.58	62.34 ± 5.69	0.000
GLS-% (mean ± SD)	18.56 ± 2.59	16.80 ± 2.63	0.000
LASr% (mean ± SD)	39.31 ± 7.27	37.59 ± 8.71	0.000
LAScd-% (mean ± SD)	25.40 ± 5.63	24.46 ± 7.07	0.001
LASct-% (mean ± SD)	19.54 ± 5.36	17.33 ± 6.48	0.000
Aortic root cm (mean ± SD)	2.93 ± 0.39	2.88 ± 0.38	0.05
LA dimension cm (mean ± SD)	3.10 ± 0.42	3.17 ± 0.44	0.000

*LASr* Left atrial strain reservoir; *EF* Ejection fraction; *LAScd* Left atrial strain conduit, *GLS* Global longitudinal strain; *LASct* Left atrial strain contraction; *LVEDD* Left ventricle end diastolic diameter; *LVESD* Left ventricle end systolic diameter; *LA* Left atrium

Significant *p* value < 0.05

**Table 3:** Correlation between different LAS measures and (LV GLS, LA dimensions) after chemotherapy

Parameters	GLS		LA dimensions	
	Spearman's rho	*p* value	Spearman's rho	*p* value
LASr1	0.315	0.002*	0.194	0.061
LAScd1	0.259	0.012*	0.151	0.145
LASct1	0.220	0.033*	0.064	0.538

*LAS* Left atrial strain, *LV GLS* Left ventricular global longitudinal strain, *LA* Left atrial; *LASr* Left atrial strain reservoir, *LAScd* Left atrial strain conduit; *LASct* Left atrial strain contraction

*Statistically significant

**Table 4:** Multivariate linear regression analysis for independent predictors of LASr after chemotherapy

	Odd's ratio	P
LVEDD	0.072	0.459
LVES	− 0.167	0.114
EF	− 0.068	0.492
GLS	− 0.124	0.277
Aortic dimension	− 0.175	0.082
LA dimension	0.025	0.803
Age	0.001	0.992
DM	− 0.162	0.273
Hypertension	0.075	0.614
Group	0.576	0.000

*EF* Ejection fraction; *LASr* Left atrial strain (reservoir); *GLS* Global longitudinal strain; *LVEDD* Left ventricle end diastolic diameter; *LVESD* Left ventricle end systolic diameter; *LA* Left atrial; *DM* Diabetes mellitus

**Table 5:** Comparison between ACEI and non-ACEI-pretreated subgroups regarding LAS and GLS measures after chemotherapy

	ACEI subgroup	Non-ACEI subgroup	*P*
	(*n* = 43)	(*n* = 51)	
LASr% (mean ± SD)	40.72 ± 5.77	38.10 ± 8.20	0.000
LAScd-% (mean ± SD)	26.15 ± 4.62	24.76 ± 6.32	0.004
LASct-% (mean ± SD)	20.90 ± 4.60	18.38 ± 5.70	0.000
GLS-% (mean ± SD)	19.38 ± 2.40	16.46 ± 2.01	0.000

*ACEI* Angiotensin-converting enzyme inhibitors; *LASr* Left atrial strain reservoir; *LAScd* Left atrial strain conduit; *GLS* Global longitudinal strain; *LASct* Left atrial strain contraction

**Table 6:** Comparison between left atrial strain measures in the ACEI subgroup

	ACEI subgroup		*P*
	(*n* = 43)		
	Before chemotherapy	After chemotherapy	
LASr% (mean ± SD)	40.75 ± 5.75	40.72 ± 5.77	0.129
LAScd-% (mean ± SD)	26.16 ± 4.64	26.15 ± 4.62	0.278
LASct-% (mean ± SD)	20.92 ± 4.62	20.90 ± 4.60	0.340

*ACEI* Angiotensin-converting enzyme inhibitors; *LASr* Left atrial strain reservoir; *LAScd* Left atrial strain conduit; *LASct* Left atrial strain contraction

There was a significant difference between LAS (only reservoir and conduit measures) after receiving chemotherapy between hypertensive and non-hypertensive patients Table 7.

**Table 7:** Comparison between left atrial strain measures in hypertensive patients versus non-hypertensive after chemotherapy

	Non-hypertensive	Hypertensive	P
LASr% (mean ± SD)	37.04 ± 6.74	42.46 ± 12.71	0.021
LAScd-% (mean ± SD)	21.30 + 6.74	30.09 ± 10.99	0.035
LASct-% (mean ± SD)	14.04 ± 6.20	15.48 ± 7.08	0.566

*LASr* Left atrial strain reservoir; *LAScd* Left atrial strain conduit; *LASct* Left atrial strain contraction

## Discussion

LA, with an average size, can be impaired. Left atrial volume index (LAVI) is the main measure commonly used to evaluate the LA structure, but at the same time, it has lower sensitivity for prompt diagnosis of LA dysfunction [14, 23]. The anteroposterior dimension (AP) was widely used, being the most consistent measurement. Relying solely on the AP linear dimension to measure LA size is not advisable because LA dimensions do not change symmetrically during the remodeling process [24, 25]. Since standard echocardiographic parameters are still normal, LAS remains a substantial marker of LA dysfunction [26] and an early indicative of diastolic dysfunction (DD) [27].

The study's results confirmed our hypothesis that decreased LAS would be a valuable and early predictor of subclinical LV systolic or DD in patients receiving anthracycline therapy. All LAS measures were significantly reduced after anthracycline therapy and LV global longitudinal strain. A significant positive correlation existed between LAS measures and LV global longitudinal strain. Left ventricular EF decreased significantly with a substantial increase in LA dimensions, but these changes were not clinically significant because all measures were within normal range. Our results agreed with Laufer et al. [28]. They found a substantial decrease in LA (reservoir) strain LASr and LA (conduit) LASc strain that occur early in anthracycline therapy, significantly correlating with standard echocardiographic diastolic measures. Our study findings were consistent with those of Emerson et al. They established a considerable decrease in LA strain after anthracycline therapy with moderate correlation with LV diastolic measures. The reduction in LA strain measures was substantial even in individuals with maintained LV systolic and diastolic functions [29].

Li et al. found promising results regarding the possible role of LA strain in the early detection of cancer therapy-related cardiac dysfunction. Eighty patients treated with anthracyclines had a relative decrease in LAS > 19% with 71.4% sensitivity and 87.9% specificity [30]. Inoue et al. also found that a decrease in LASr 3 months following anthracycline therapy was independently associated with later cardiotoxicity [31]. We preferred to assess LV global longitudinal strain as an index of subclinical LV dysfunction that occurs early before evident LV dysfunction with the decline of EF below the normal range, i.e., less than 50%. The decrease in LV global longitudinal strain is expected to contribute to DD, leading to the deterioration of LAS measures. A significant positive correlation between LAS measures and LV global longitudinal strain confirmed this theory. A subgroup of hypertensive patients on ACEI was protected against deterioration of LAS measures; however, the LV global longitudinal strain reduction was significant. This was not of clinical significance because LV global longitudinal strain remained within the normal range in this subgroup of patients. This indicates the potential cardioprotective effect of ACEI in patients receiving anthracycline therapy. Ayuna et al. concluded that neurohormonal blockers like ACEI and beta-blockers are indicated to prevent early cardiotoxicity [32]. Among the many other applications, the protective effects of ACEIs on the heart have been one of the major areas of study concerning cardio protection. More recently, prevention of heart failure and myocardial infarction was noted with ACEIs. Wu et al. conducted a meta-analysis in 2023, which proved that ACEIs decrease the incidence of heart failure in high-risk groups, presenting their efficiency in primary prevention challenges. This cardioprotection is associated with the reduction of malicious cardiac remodeling and improvement in endothelial function, both vital in maintaining cardiovascular health [33]. As for secondary prevention, ACEIs have been shown to reduce morbidity and mortality post-acute coronary syndrome. Chen et al. (2023) established in their controlled randomized study that patients with myocardial infarction treated with ACEIs had a 30% reduced risk of recurring adverse cardiovascular events compared to patients not subjected to ACEI treatment. The benefits are believed to emanate from the reduced cardiac afterload and improved myocardial oxygen delivery, which are important factors in the recovery process after myocardial infarction. This study also illustrated the need for early initiation of ACEI to bring about better long-term outcomes [34]. The heart-protective benefits of ACEIs extend to patients with other comorbidities like diabetes and hypertension. The benefits of ACEI in blood pressure reduction and improving renal outcomes, thereby reducing cardiovascular risk in diabetic patients, were replicated in a 2023 study conducted by Patel et al. [35]. This dual advantage underlines the critical role of ACEIs in the systemic management of cardiovascular risk, especially in multiple-risk-factor populations. Generally, these findings confirm the further use of ACEIs in both primary and secondary prevention strategies and position them as one of the cornerstones in heart protection.

Considering the small size of our study, the clinical implications of using LAS as an early marker of cardiotoxicity can be further extended. For patients with diastolic heart failure, lost atrial contraction in sinus rhythm patients had a worse prognosis than patients with sinus rhythm and preserved atrial contraction [36]. In patients with diabetes or hypertension, LAS may be a sensitive method for detecting patients at risk for the development of LA enlargement and its complications; therefore, it may help choose patients who need strict glucose and blood pressure control. Decreased LAS also had increased future risk of atrial fibrillation and stroke risk. LAS can form a valuable method for the risk stratification of patients with significant valvular cardiac disease besides indicating the optimal timing for intervention [37].

### Study limitations

The study was observational and non-randomized with a small number of patients. We did not follow the patients for more extended periods following anthracycline therapy. We did not observe the cardioprotective effect of ACEI for primary prevention in normotensive patients. 56.4% of enrolled patients were hypertensive. Hypertension is a major predictor of diastolic dysfunction and atrial strain. However, there was a significant correlation between different atrial strain measures and global longitudinal systolic strain of the left ventricle. The unity of antihypertensive medications and the presence of different comorbidities can be an additional confounding factor. However, there was a considerable difference between ACEI and non-ACEI-pretreated patients (including those who are not hypertensive) regarding LAS measures. The specific variables, including non-hypertensive patients, other chemotherapeutic agents, and other types of cancer, should be considered in future research to assess the cardioprotective role of ACEIs during chemotherapy. A combination of all other guidelines-directed medical heart failure therapy can be potential variable to be addressed in future studies.

## Conclusion

Deterioration of LAS measures is significant after anthracycline therapy and may be an early predictor of chemotherapy-induced cardiotoxicity. Pretreatment with ACEI may be protective against anthracycline-induced cardiotoxicity. ACEIs in patients who receive anthracycline should be studied in larger randomized controlled trials with extended periods of follow-up for possible primary prevention to protect from cardiotoxicity.

## Data Availability

The datasets used and analyzed during the current study are available from the corresponding author upon request.

## Abbreviations

LAS: Left atrial strain
ACEI: Angiotensin-converting enzyme inhibitors
EF: Ejection fraction
FS: Fractional shortening
LV EF: Left ventricular ejection fraction
STI: Speckle tracking imaging
MRI: Magnetic resonance imaging
LAS: Left atrial strain
LA: Left atrium
LASr: Reservoir phase
LAScd: Conduit phase
LASct: Pump phase
GLS: Global longitudinal strain
LAVI: Left atrial volume index
DD: Diastolic dysfunction (DD) setting
AP: Anteroposterior
